# Early prediction of therapy response in patients with acute myeloid leukemia by nucleosomal DNA fragments

**DOI:** 10.1186/1471-2407-6-143

**Published:** 2006-05-30

**Authors:** Susanne Mueller, Stefan Holdenrieder, Petra Stieber, Torsten Haferlach, Andreas Schalhorn, Jan Braess, Dorothea Nagel, Dietrich Seidel

**Affiliations:** 1 Institute of Clinical Chemistry, University Hospital Munich-Grosshadern, Marchioninistr. 15, D-81377 Munich, Germany; 2 Department of Internal Medicine III, University Hospital Munich-Grosshadern, Marchioninistr. 15, D-81377 Munich, Germany

## Abstract

**Background:**

Elevated levels of nucleosomal DNA fragments can be detected in plasma and sera of patients with malignant diseases.

**Methods:**

We investigated the course of nucleosomal DNA, thymidine kinase, lactate dehydrogenase and leukocytes in sera of 25 patients with acute myeloid leukemia during the first cycle of induction chemotherapy and tested their power to distinguish between patients with complete remission and those with no remission.

**Results:**

Almost all patients showed strongly decreasing levels of nucleosomal DNA during the first week, in some cases after initial peaks. In overall analysis of variance, DNA levels could clearly distinguish between patients with complete remission, who had higher DNA values, and those with insufficient response (p = 0.017). The area under the curve of DNA values of days 2–4 after start of therapy (AUC 2–4) discriminated between both groups with a sensitivity of 56% at a specificity of 100%. Further, pretherapeutic levels and AUC 2–4 of nucleosomal DNA correlated significantly with blast reduction after 16 days. A tendency to higher levels in patients with complete response was also found for thymidine kinase, lactate dehydrogenase and leukocytes, however the difference did not reach the level of significance (p = 0.542, p = 0.260, and p = 0.144, respectively).

**Conclusion:**

Our results indicate that nucleosomal DNA fragments are valuable markers for the early prediction of therapeutic efficacy in patients with acute myeloid leukemia.

## Background

The incidence of acute myeloid leukemia (AML) has remained stable over the last decades and has averaged between 1998 and 2002 at about 3.8 per 100,000 persons and year. The age-adjusted incidence rates showed a distinct difference between patients under 65 years with 1.8 per 100,000 and those over 65 years with 17.9 per 100,000 [1]. With regard to the treatment of AML, a standardized procedure has been established including induction chemotherapy to eliminate the blasts and to achieve remission followed by a consolidation therapy to stabilize the remission status [2,3]. Unfortunately the rate of complete remission after induction therapy, particularly in older patients, ranges only between 38% and 62% and could not been improved during the recent years. Younger patients often receive more intensive treatment such as double induction therapies including TAD/TAD (TAD: cytarabine, daunorubicine, 6-thioguanine) or TAD/HAM (HAM: cytarabine, mitoxantrone) and complete remission is achieved in 65% to 71% [3,4] of these cases.

Besides age there are several prognostic markers for AML patients that are used in clinical routine: Cytogenetics contain the most important prognostic information [5-7], as does the blast number in the bone marrow: High amounts of bone marrow blasts before therapy are associated with adverse outcome [8] as well as incomplete elimination during the course of chemotherapy [9]. Lactate dehydrogenase (LDH) and leukocyte number are further markers which are relevant for prognosis [6,8]. However, additional and more precise markers are warranted to estimate the therapy response as early as possible.

Chemotherapeutic agents induce apoptotic cell death in target cells like cells of tumorous tissue [10-13]. The apoptotic process by cytoxic drugs is started by direct damage of DNA, interference in the cell cycle [10,14], or by the activation of apoptotic receptors such as Fas/Apo1/CD95 [11,12]. Subsequently a cascade of enzymes is activated [15] which results in the degradation of the cellular structure and cleavage of the chromatin into mono- and olignucleosomes by various caspases and nucleases [16-18]. These nucleosomes are packed into apoptotic bodies and are engulfed by neighbouring cells and macrophages. However a substantial part is released into circulation particularly in high rates of cell death [19,20]. Thus, nucleosomal DNA levels in plasma and serum can be correlated with the extent of cell death at a specific time point. Measurement of circulating DNA fragments can be performed in plasma and serum by various methods including real time PCR and ELISA [21,22].

Healthy individuals and patients with benign diseases show lower levels of nucleosomal DNA in their circulation than persons with malignant diseases [22-27]. In previous investigations typical kinetics of nucleosomal DNA could be observed during radio- and chemotherapy [28]. Patients with solid tumors receiving chemotherapy showed a rapid increase of nucleosomal DNA levels 24–72 hours after the application followed by a decrease to reach the basal levels after about one week [25,27]. With regard to the early prediction of therapeutic efficacy, patients with lung cancer with insufficient response to chemotherapy showed a higher increase and a less effective decrease of nucleosomal DNA values during the first week than those with good response to therapy [29].

In contrast to solid tumors, malignant leukemic cells are always present in the bone marrow and/or in the blood circulation. Thus the release of nucleosomal DNA into serum or plasma is facilitated and is independent of the blood supply of the tumor.

The present study was undertaken to investigate the courses of nucleosomal DNA fragments, thymidine kinase, lactate dehydrogenase and leukocytes in patients with AML during chemotherapy. The main purpose was to explore whether the courses of nucleosomal DNA in AML patients differ from those of patients with solid tumors and whether they could early and reliably predict the response of AML patients treated with standard chemotherapy.

## Methods

### Patients

Twenty five consecutive patients with acute myeloid leukemia (AML) were included in our study, among them 23 patients with de novo AML and 2 patients with relapse of the disease.

The de novo AML patients received induction chemotherapy with one or two cycles of the TAD and/or HAM-protocols. TAD chemotherapy, which generally was applied as first induction course, contained 100 mg/m^2 ^cytarabine (days 1, 2 and 3 through 8 every twelve hours), 60 mg/m^2 ^daunorubicin (days 3–5) and 100 mg/m^2 ^6-thioguanine (days 3–9 every twelve hours). HAM was given as second cycle or as first and second induction course consisting of 3 g/m^2 ^(<60 years) or 1 g/m^2 ^(>60 years) cytarabine (days 1–3 every twelve hours) and 10 mg/m^2 ^mitoxantrone (days 3, 4). The two patients with relapsed AML received a cytarabine + daunorubicin combination and the F-S-Hai-protocol, respectively. Characteristics of the patients are summarized in table 1. [Table 1]

The study was approved by the local ethics committee. Before inclusion in the study, written informed consent was given by all patients.

### Estimation of treatment efficacy

With regard to therapy response, it was differentiated between patients with complete remission (n = 18) and those with failure (n = 7) of therapy. According to the criteria of the German AML Cooperative Group, complete remission was defined by bone marrow cytology performed after induction therapy, where there were less than 5% blasts and normal hematopoiesis of all cell lines in the bone marrow, and more than 1,500 neutrophils and 100,000 platelets per microliter in peripheral blood for at least four weeks. Patients with insufficient decline of the blasts, death earlier than 7 days after the end of the first induction cycle or death because of the treatment-induced bone marrow hypoplasia after chemotherapy were categorized as therapeutic failure [4].

### Measurement of blood parameters

Blood samples were taken before the first application of chemotherapy (day 1) and then daily in the morning during the whole cycle. Preanalytical handling of serum samples was performed as described earlier [22]: Blood samples were centrifuged at 3000 g for 15 minutes, treated with 10 mM EDTA immediately after the centrifugation and stored at -80°C. Subsequently, the samples were thawed and nucleosomes were measured in batches combining all samples of a patient to minimize the interassay variability by the Cell Death Detection-ELISA^plus ^of Roche Diagnostics. The samples were placed into a microtiterplate and, additionally, a mixture of anti-histone-antibodies, anti-DNA-antibodies and a buffer solution: Anti-histone-antibodies bound to the histone component and were fixed to the microtiterplate whereas anti-DNA-antibodies labelled with peroxidase recognized the DNA component of the nucleosomes. After a washing step, the peroxidase retained in the immunocomplex reacted with 2,2'-azino-di(3-ethylbenzthiazoline-sulfonat) substrate and the amount of nucleosomes was determined quantitatively by spectrophotometrical analysis in ng/mL DNA.

In parallel, thymidine kinase (TK) was measured by a radioimmunoassay (Immunotech), leukocytes and lactate dehydrogenase (LDH) were determined daily by routine methods (Coulter LH 750 Analyzer, Beckman, and Olympus AU 2700, Olympus, respectively) on the day of sample collection.

### Statistics

Correlations between nucleosomal DNA fragments and leukocytes on the one hand and blast number on the other hand were calculated by Spearman rank correlation.

An overall analysis of variance was performed to test the general dependency of the concentrations of DNA fragments, thymidine kinase, lactate dehydrogenase and leukocytes upon response to therapy (complete response versus no response) and on time before (day 1) and after start of chemotherapy (day 2 – day 8), respectively. In case of a significant overall effect of therapy, the effects of single days were tested, too. Additionally, the interaction between therapy response and time was investigated. This was done using SAS Procedure MIXED, which can take into account dependencies of repeated values of the same patient. For this analysis data were used as logarithms.

Using Wilcoxon test, the marker levels, their changes from pretherapeutic value (day 1) to day 2 and the area under the curve of nucleosomal DNA values from day 2 to 4 after start of therapy (AUC 2–4) were analyzed on their discriminative power between the two response groups. To calculate AUC 2–4, the concentrations of days 2, 3 and 4 were mandatory.

For all evaluations, a p-value <0.05 was considered statistically significant. All calculations were performed by software of SAS (version 8.2, SAS Institute Inc., Cary, NC, USA).

## Results

Eighteen patients out of twenty five showed a complete remission in the peripheral blood and bone marrow after induction or relapse chemotherapy. The remaining seven AML patients showed no or only insufficient response to therapy and four of them died during the bone marrow hypoplasia after treatment.

During the first week of chemotherapy, a decrease of nucleosomal DNA levels was observed in almost all patients. In some patients, DNA concentrations increased temporarily immediately after start of the therapy. 50% of the patients in the remission group (9 of 18) showed an early increase at day 2, whereas values decreased immediately in most non-responsive patients (6 of 7). However, there was no significant difference between both groups concerning the percentual changes from day 1 to 2 (p = 0.259). [Table 2]

**Figure 1:**
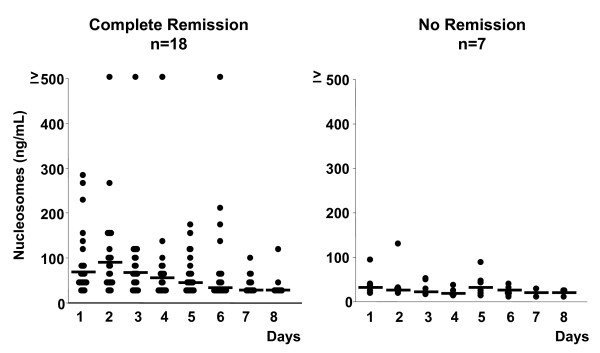
Distribution of nucleosomal DNA in patients with complete remission and no remission during the first week of induction therapy (-- = medians).

**Figure 2:**
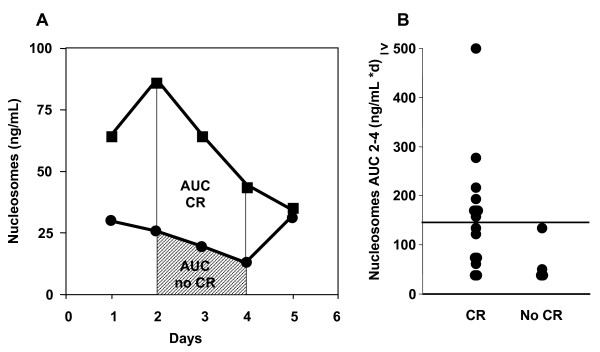
The area under the curve of nucleosomal DNA values of days 2–4 of induction therapy (AUC 2–4) discriminates between patients with complete remission and those with no remission. **A) **AUC 2–4 of nucleosomal DNA is significantly larger for patients with complete response (CR ■) than for those not responsive (no CR ●) to therapy (p = 0.042). Here, median levels of days 1 to 4 and the respective AUC 2–4 are shown for both response groups. **B) **Distribution of AUC 2–4 levels of nucleosomal DNA. Favourable therapy response can be predicted with a sensitivity of 56% and a specificity of 100% at a cutoff level of 150 ng/mL*d.

Generally, higher values were measured in patients who achieved complete remission compared with those who showed an insufficient therapy response [Figure 1]. In overall analysis of variance, levels of nucleosomal DNA fragments were dependent on both variables being therapy response (p = 0.017) and time after start of chemotherapy (p = 0.023), respectively. No interaction was observed between therapy response and time (p = 0.495). In detailed analysis, significantly higher values of nucleosomal DNA were found for the days 2 and 4 (p = 0.014 and p = 0.022, respectively); day 3 was of borderline significance (p = 0.051). If these values were integrated in the area under the curve of days 2–4 (AUC 2–4) of circulating nucleosomal DNA, a significant difference between patients with complete response and those who were not responsive to therapy was observed (p = 0.042). By AUC 2–4, both groups could be separated with a sensitivity of 56% and a specificity of 100% at a cutoff level of 150 ng/mL*d [Figure 2].

Thymidine kinase levels increased steeply from day 1 to day 4 followed by a rapid decrease. In analysis of variance, thymidine kinase levels were only dependent on time after start of chemotherapy (p = 0.001), but not on therapy response (p = 0.750), though the median levels at single days were higher in patients with complete remission. There was also no interaction between therapy response and time (p = 0.360). [Table 2]

Levels of lactate dehydrogenase were neither dependent on time after start of chemotherapy (p = 0.161), nor on therapy response (p = 0.251). There was also no interaction between therapy response and time (p = 0.359). [Table 2]

Leukocyte levels remained almost constant until day 3 in patients with complete response whereas they decreased immediately after start of chemotherapy in most of the patients with insufficient response. In analysis of variance, leukocytes showed a clear dependency on time after start of chemotherapy (p < 0.001), but not on therapy response (p = 0.193). There was also no interaction between therapy response and time (p = 0.914). However, with respect to the percentual changes from day 1 to 2, a borderline significant difference was observed (p = 0.059). [Table 2]

Investigating the immediate effect of therapy on markers concentrations, a positive correlation was identified for the levels of nucleosomal DNA fragments at day 2 with leukocytes at day 2 (r = 0.54; p = 0.009) as well as with the increase of leukocytes from day 1 to 2 (r = 0.58; p = 0.010). This correlation was maintained in the subgroup of patients with complete remission (r = 0.76; p = 0.001 and r = 0.56; p = 0.049, respectively), but not in the subgroup of patients with insufficient response (r = 0.20; p = 0.704 and r = -0.31; p = 0.544, respectively).

Because cytogenetics and immunophenotype were very heterogeneous, correlations with therapy response were not performed. The bone marrow blast numbers at days 1 and 16 as well as their percentual changes were comparable in both response groups and could not discriminate between patients with complete remission and those with no response to therapy. [Table 2]

No correlation was observed between pretherapeutic nucleosomal DNA levels and bone marrow blast number (r = 0.08; p = 0.722). However, pretherapeutic nucleosomal DNA levels correlated inversely with bone marrow blast number after 16 days (r = -0.55; p = 0.012) and with the relative reduction of blast number from day 1 to 16 (r = 0.58; p = 0.007). Similarly, the area under the curve of days 2–4 (AUC 2–4) of circulating nucleosomal DNA reflecting the immediate effect of therapy correlated with the reduction of blast number from day 1 to 16 (r = 0.49; p = 0.041).

## Discussion

Treatment of acute myeloid leukemia aims to achieve complete remission. In contrast to solid tumors which show a response to therapy when there is a reduction of the tumor mass, patients with AML are considered sufficiently treated only when there are no blasts in the peripheral blood or less than 5% blasts in the bone marrow. The AML patients receive systemic chemotherapy partitioned to at least two parts: the induction therapy aiming at effective reduction of malignant cells and the consolidation therapy consisting of chemotherapy with or without autologous or allogeneic stem-cell transplantation to prevent relapse of leukemia [2,3].

Antileukemic drugs like cytarabine in combination with daunorubicine and 6-thioguanine (TAD), or with mitoxantrone (HAM) cause DNA damage or inhibit DNA synthesis and, thus, kill tumor cells mainly by apoptosis [30]. The activation of caspases-3 and caspases-8 was already detected 12–24 hours after the application of the chemotherapeutic drugs [31,32]. Also morphologically, increasing numbers of apoptotic cells were observed after application of the cytotoxic therapy; for example mitoxantrone induced early apoptosis and showed a high rate of cell death of blast cells during the initial 24 hours [14]. Another study reported cytarabine to induce cytotoxicity and to reduce the viability of bone marrow blasts by two thirds 24 hours and almost completely 96 hours after application of the drug [32].

At high rates of spontaneous and treatment-induced apoptosis, tumor cell-free DNA was measured in blood circulation by immunoassays or by real time PCR [21,22]. A plentitude of studies report higher levels of cell-free DNA in plasma and serum of cancer patients than in healthy individuals regarding almost all types of solid tumors, leukemia or lymphoma [23-27,33-37]. During the course of anticancer therapy in patients with solid tumors, levels of cell free DNA decreased in patients who showed effective response to therapy whereas they increased or remained constant in those patients with stable or progressive disease or at time of relapse [23,38-40]. The same effects were observed in nasopharyngeal carcinomas and lymphomas during radiotherapy or chemotherapy concerning EBV-DNA [41-43].

Most DNA in circulation is supposed to be organized in nucleosomes as complexes of DNA and histones [20,44-46]. As protein bound particles, they seem to be better conserved against rapid digestion by serum and plasma endonucleases [47]. Methods for the detection of cell free DNA and nucleosomal DNA have shown good correlations with regard to single values as well as to serial measurements [48]. During the initial phase of chemotherapy, levels of nucleosomal DNA showed a rapid increase in the blood of patients with solid tumors already 24 – 72 hours after application followed by a decrease during the first week [25,27,29]. In various solid tumors, the kinetics of the nucleosomal DNA levels during chemo- and radiotherapy correlated with the therapy response: Declining baseline values determined before the various cycles were associated with effective treatment, whereas stable or increasing values were observed in patients with insufficient response [25,28,29]. In addition, the early changes of nucleosomal DNA levels have proved to be valuable for the early prediction of the therapeutic efficacy in patients with lung cancer: Patients with remission showed only minor increases of nucleosomal DNA followed by rapid and complete decreases during the first week of chemotherapy. Patients with progressive disease, however, had stronger increases and less complete decreases of nucleosomal DNA. Thus, the resulting area under the curve from day 1 to 8 was able to predict the later treatment response [29]. In pancreatic cancer during radiotherapy too, response was anticipated by changes of nucleosomal DNA levels already during the first days of the therapy [50].

In this study, we investigated the concentration of nucleosomal DNA fragments in patients with AML daily during the first week of induction therapy. The serum values decreased significantly in all patients during this time frame. However, in patients who later achieved complete remission, levels remained constant or increased only slightly on day 2 followed by a rapid and continuous decrease to approximately one third of the pretherapeutic level. Generally, patients with response tended to have higher nucleosomal DNA levels than non-responding patients. These changes of serum nucleosomal DNA in AML patients contrasted clearly to our earlier results in patients with solid tumors: In patients with AML, higher nucleosomal DNA levels were detected in those who were responsive to therapy, whereas in patients with solid tumors they were higher in the non-responsive group. These controverse observations might be explained by different pathophysiological backgrounds of the tumor entities:

Patients with solid tumors treated with systemic therapies often have aggressive tumor types in already advanced stages with high tumor load. The high levels of circulating nucleosomal DNA particularly in those patients who are non-responsive to antitumor therapy might be due to a) a higher rate of cellular turnover before and during therapy corresponding with a higher release of nucleosomal DNA, b) a higher rate of dysfunctional cells easily killed by cytotoxic therapies, c) a better blood supply of the tumor tissue facilitating the transition of nucleosomal DNA into blood circulation, and d) a defective clearance system of circulating nucleosomal DNA. Though in both, responsive and non-responsive patients with solid tumors, a considerable number of tumor cells will die during therapy, the effective elimination of nucleosomal DNA in blood and already before transition into circulation is supposed to be associated with a more functional immune system in patients with less advanced tumors and better therapy response.

In AML, malignant blasts are present in the blood circulation and bone marrow and constitute there the target for chemotherapeutic agents. This effect was shown by flow immunocytometric methods that detected high numbers of apoptotic peripheral blood lymphocytes and blasts in the circulation shortly after application of cytotoxic chemotherapy depending on the type and dose of the drugs [14,51]. High rates of apoptotic cells and, thus, high levels of nucleosomal DNA, passing directly over into blood circulation, correspond with sufficient response to the respective treatment whereas low levels correlate with relative resistance of target cells to undergo apoptosis. As nucleosomal DNA is already released 12–24 hours after the apoptotic event [52], and as they are removed rapidly from circulation under physiological conditions, the early increase followed by a rapid decrease during the induction therapy might reflect the effective elimination of leukemic cells [53,54].

As well as malignant cells, other rapidly proliferating cells are also damaged by cytotoxic drugs resulting in side effects such as myelotoxicity including anemia, leukopenia and thrombopenia as well as mucositis and gastrointestinal symptoms. In addition, specific side effects are known to be caused by some drugs such as pulmonary toxicity for cytarabine and cardiotoxicity for daunorubicine and mitoxantrone. Besides leukemic cells, therefore, also other normal or inflammatory cells might contribute to the release of nucleosomal DNA.

To specify the information given by circulating nucleosomal DNA, we compared it with the number of leukocytes and already established proliferation and cell death markers such as thymidine kinase and lactate dehydrogenase, which are already in clinical use for patients with leukemia or lymphomas. In recent studies, AML patients with a high pretherapeutic thymidine kinase activity have often achieved an adequate blast clearance after the first induction cycle [55]. Additionally in agreement with this data, levels of thymidine kinase tended to be higher in patients with complete remission pretherapeutically as well as during the whole first cycle in our study. Similarly, we observed somewhat higher lactate dehydrogenase concentrations in patients with complete remission during chemotherapy. Though there was strong dependency of thymidine kinase and LDH concentrations on time after start of chemotherapy, there was no significant association with the response to therapy for both markers.

Remarkably however, the levels of nucleosomal DNA fragments were clearly dependent on both time after start of chemotherapy and therapy response, respectively, whereas treatment efficacy was not related to time. The most significant differences between both response groups were found in particular for nucleosomal DNA levels at the very initial phase of the therapy. This observation reflects the constantly high DNA concentrations during days 1 to 4 in patients with complete remission in contrast to the early declining levels in those patients with insufficient response. An elegant parameter to integrate the information given at the single days is the area under the curve of days 2–4 (AUC 2–4) of nucleosomal DNA. As the pretherapeutic value was not considered in this variable, AUC 2–4 reflected the immediate therapy effect on the release of nucleosomal DNA. Consistently with the results of the single days, AUC 2–4 of nucleosomal DNA could discriminate between the groups of patients with complete response and those who were not responsive to therapy (p = 0.042). In particular, AUC 2–4 enabled the prediction of favourable therapy response with a sensitivity of 56% at a specificity of 100%.

High values in responsive patients and early decreases in non-responsive patients were observed for leukocytes, too. Although there was no significant difference in the absolute leukocyte counts between both groups, the relative changes from day 1 to 2 were of borderline significance (p = 0.059).

Most notably, the correlation analysis revealed an interrelationship of leukocytes on day 2 or changes of leukocyte numbers during the course of therapy with the concentrations of DNA fragments in serum (p = 0.009 and p = 0.010, respectively). This observation suggests that leukocytes might be an important source or at least a stimulator for the release of DNA fragments into blood or they might prevent their elimination from circulation. As this correlation was particularly relevant for patients with complete remission but not for those with failure to therapy, it seems reasonable that the higher levels of circulating nucleosomal DNA fragments are mainly the result of effective blast reduction. This hypothesis is further strengthened by our observation that the pretherapeutic levels and the AUC 2–4 of nucleosomal DNA, reflecting the immediate effect of therapy, were related to the percentual reduction of bone marrow blast number from days 1 to 16.

The correlation of further parameters which are used in clinical routine for diagnostic or prognostic purposes, such as cytogenetics and immunophenotype, with nucleosomal DNA and therapy response was not possible in our setting as the patient number was limited and the heterogeneity of these markers was considerable. However, it would be valuable to include these markers together with the kinetics of circulating nucleosomal DNA fragments in prospective trials to elucidate their potential additive power for the early prediction of therapy response.

## Conclusion

Our results indicate that, in AML patients, the changes of nucleosomal DNA during the initial phase of induction chemotherapy are valuable markers for the early estimation of therapy response and should be validated in further prospective trials.

## Competing interests

The author(s) declare that they have no competing interests.

## Authors' contributions

SM, SH, PS, DN and DS designed the present study and coordinated its logistic process. SM, TH and AS were responsible for the recruitment of the patients and the defined blood drawings. SM carried out the immunoassays. TH and JB did the investigations on immunophenotype, cytogenetics and blast numbers. DN performed the statistical analysis. SM, SH, PS, DN and DS were  involved in the interpretation of the data and the conception of the manuscript. All authors read and approved the final manuscript.

## Pre-publication history

The pre-publication history for this paper can be accessed here:



## Figures and Tables

**Table 1:** Characteristics of the patients investigated

	Median	Range
Age (y)	62	22 – 76
	Number	Percentage

Gender		
Female	12	(48.0)
Male	13	(52.0)

Histology		
M1	6	(24.0)
M2	8	(32.0)
M3	1	(4.0)
M4	5	(20.0)
M6	3	(12.0)
Unknown	2	(8.0)

Cytogenetics		
Normal	13	(12.0)
Trisomy 8	1	(4.0)
Trisomy 11	1	(4.0)
Trisomy 21, Isochrom X	1	(4.0)
Mosaic 46XX, 45X0	1	(4.0)
Inversion 16	2	(4.0)
Inversion 3	1	(4.0)
Inversion 3, Monosomy 7	1	(4.0)
Pericentric Inversion 4	1	(4.0)
Translocation (2;5)	1	(4.0)
Translocation (8;21)	1	(4.0)
Translocation (15;17)	1	(4.0)

Immunophenotype		
MPO, CD13, CD33	15	(60.0)
MPO, CD13, CD33, CD 14	1	(4.0)
MPO, CD13, CD33, CD3, CD7, Cd2, TdT	1	(4.0)
Not available	8	(32.0)

Mode of therapy		
TAD	9	(36.0)
HAM	5	(20.0)
TAD/HAM	7	(28.0)
HAM/HAM	2	(8.0)
Relapse therapy	2	(8.0)

Therapy response		
Complete remission	18	(72.0)
Non-Responders	7	(28.0)

**Table 2:** Correlation of nucleosomal DNA, thymidine kinase, lactate dehydrogenase, leukocytes and bone marrow blast number with response to therapy

	***Complete response***			***No response***			***P-value***
	**N**	**Median**	**Range**	**N**	**Median**	**Range**	
**Nucleosomal DNA fragments (ng/mL)**							
Effect of therapy response on nucleosomal DNA							**0.017**
Effect of time after start of chemotherapy (days) on nucleosomal DNA							**0.023**
Interaction between therapy response and time							0.495
Day 1 (before therapy)	15	**65.2**	22.3 – 267.1	7	**30.3**	16.0 – 90.9	0.104
Day 2	18	**86.4**	21.7 – 807.7	6	**25.7**	17.7 – 128.1	**0.014**
Day 3	18	**63.5**	11.4 – 657.8	6	**21.2**	12.0 – 50.3	0.051
Day 4	16	**44.6**	9.7 – 613.8	5	**14.9**	12.0 – 36.0	**0.022**
Day 5	16	**36.0**	12.6 – 154.4	6	**31.5**	12.0 – 86.4	0.530
Day 6/7/8	18	**24.0**	9.2 – 2031.2	6	**16.6**	9.2 – 38.3	0.060
Day 2 – Day 1 %	15	**2.5**	-76 – 305	6	**-16.5**	-35.3 – 40.9	0.259

**Thymidine kinase (U/L)**							
Effect of therapy response on thymidine kinase							0.750
Effect of time after start of chemotherapy (days) on thymidine kinase							**0.001**
Interaction between therapy response and time							0.360
Day 1 (before therapy)	15	**50.6**	6 – 603	7	**40.9**	10 – 1095	
Day 2	17	**88.9**	1 – 727	6	**54.1**	22 – 127	
Day 3	18	**183.0**	5 – 1789	6	**121.2**	47 – 998	
Day 4	15	**302.0**	25 – 1158	5	**85.6**	58 – 772	
Day 5	14	**194.5**	22 – 1161	6	**193.5**	57 – 758	
Day 6/7/8	18	**175.5**	29 – 1040	7	**146.0**	36 – 414	
Day 2 – Day 1 %	14	**21.9**	-90 – 626	6	**50.2**	-63 – 110	0.970

**Lactate dehydrogenase (U/L)**							
Effect of therapy response on LDH							0.251
Effect of time after start of chemotherapy (days) on LDH							0.161
Interaction between therapy response and time							0.359
Day 1 (before therapy)	10	**346**	120 – 672	5	**326**	202 – 427	
Day 2	10	**373**	177 – 1603	4	**237**	147 – 391	
Day 3	11	**361**	204 – 876	3	**278**	183 – 282	
Day 4	9	**533**	153 – 1068	3	**285**	217 – 330	
Day 5	9	**380**	182 – 1405	3	**318**	218 – 440	
Day 6/7/8	12	**304**	174 – 1087	4	**261**	232 – 336	

**Leukocytes (G/L)**							
Effect of therapy response on leukocytes							0.193
Effect of time after start of chemotherapy (days) on leukocytes							**<0.001**
Interaction between therapy response and time							0.914
Day 1 (before therapy)	14	**3.4**	1.2 – 52.5	7	**3.4**	0.7 – 37.4	
Day 2	16	**3.7**	0.5 – 43.1	6	**2.2**	0.6 – 30.3	
Day 3	16	**3.8**	0.5 – 23.0	5	**1.6**	0.9 – 17.0	
Day 4	13	**2.1**	0.6 – 22.3	3	**1.7**	0.9 – 5.4	
Day 5	11	**1.7**	0.7 – 3.4	3	**0.8**	0.7 – 1.5	
Day 6/7/8	13	**0.9**	0.5 – 21.6	3	**0.9**	0.5 – 1.1	
Day 2 – Day 1 %	13	**-1.4**	-61 – 79	6	**-35.4**	-54 – -14.3	0.059

**Bone Marrow Blast Number (%)**							
Day 1 (before therapy)	18	**72**	7 – 90	7	**74**	25 – 93	0.880
Day 16	17	**0**	0 – 6	6	**4**	0 – 72	0.385
Day 1 – Day 16 %	17	**100**	14 – 100	6	**88**	3 – 100	0.385
